# Spotify Tailoring for Architectural Governance

**DOI:** 10.1007/978-3-030-58858-8_24

**Published:** 2020-08-18

**Authors:** Abdallah Salameh, Julian M. Bass

**Affiliations:** 6 IT University of Copenhagen, Copenhagen, Denmark; 7 University of British Columbia, Vancouver, BC Canada; University of Salford, 43 Crescent, Salford, M5 4WT UK

**Keywords:** Spotify tailoring, Architecture governance, Autonomous teams, Large-scale, FinTech, Intervention embedded case study

## Abstract

Organisations usually tailor Agile methods to fit their needs best. Spotify has developed its own Agile culture to facilitate software development for hundreds of developers across multiple cities. The Spotify model has become influential among agile proponents and hence formed the basis of methods used in other organisations. We have identified a lack of research into agile architecture using the Spotify model.

To explore *How can architectural governance increase the autonomy of teams when using the Spotify model?*, an intervention embedded case study was conducted in a multinational FinTech organisation, using the Spotify model. New processes were introduced by developing and evaluating an approach to Agile architectural governance. This approach incorporates a structural change and a change management process. We conducted 6 semi-structured open-ended interviews and direct observations of Agile practices. The collected data was analysed using Thematic Analysis and informed by some Grounded Theory techniques.

The practitioners in our study report benefits of this evaluated approach. These benefits include transforming architectural based decision into decentralised based decision-making, strengthening the autonomy of squads through aligning architectural based decisions, sharing the architectural knowledge among the squads, and other benefits.

We identify the characteristics and benefits of our evaluated approach to Agile architectural governance using the Spotify model. Also, we identify guidelines and challenges for those wishing to adopt this approach.

## Introduction

The introduction of agile software development has shifted the focus from the individual level into the team level by employing self-organising autonomous teams [8]. These teams should be aligned with each other and to common product development objectives to enable their autonomy [9]. Previous research has identified the topic of autonomous teams as immature within software engineering because of some identified challenges that need addressing [12].

The Spotify model is an example of an agile approach that is driven by creating autonomous, yet aligned squads (i.e., teams) [6]. In our previous work on Spotify Tailoring, we have identified tailored practices that promote effectiveness in autonomous squads [9] and have revealed a novel approach to agile tailoring using the Spotify model, which we called Heterogeneous Tailoring [11]. Two key features characterise this approach. Firstly, each autonomous cross-functional squad is empowered to select and tailor its development method. Secondly, each squad is aligned with other squads and to common product development goals.

One of the identified challenges to the Heterogeneous Tailoring approach is the need for aligning and governing architectural decisions across autonomous squads [11]. In the same way, previous research on the topic of autonomous teams has identified the coordination of system architecture among autonomous teams as a challenge that needs exploration [12]. Often, positions are linked to individuals, which leads to a bottle neck since one person bears many responsibilities and has to reply to many different demands simultaneously [4]. External dependencies to one person decreases team autonomy and its innovation. We found that our case study organisation utilises a centralised architectural decision-making while using the Spotify model.

Our research question in this study is: *How can architectural governance increase the autonomy of teams when using the Spotify model?* To answer this question, we have conducted an intervention embedded case study in a multinational FinTech organisation uses the Spotify model with a large-scale project. In this intervention, we develop and evaluate an approach to architectural governance. In this approach, we introduce specific roles in each squad to increase autonomy within squads instead of relying on one person (i.e., architect) who is responsible for all teams. Also, we introduce a change management process to streamline the agile architecture process among the stakeholder. During this intervention trial, we have conducted a direct observation of 23 ceremonies over 9 weeks. After that, 6 semi-structured interviews were conducted.

The practitioners in our study report benefits of this approach such as transforming architectural decision-making into decentralised based decision-making, resolving conflicted architectural decisions and mitigating key technical risks across autonomous squads, strengthening the autonomy of squads through aligning architectural based decisions, and other benefits.

In this paper, we identify the characteristics and benefits of our evaluated approach to architectural governance. Also, we identify guidelines and challenges for those wishing to adopt this approach using the Spotify model.

## Background

### The Spotify Model

The Spotify model, which was introduced by Henrik Kniberg [6, 9], has been developed to utilise agile development with hundreds of developers that are distributed among many squads and across 4 cities. The overall structure consists, mainly, of Squads, Chapters, Guilds and Tribes. A Tribe is a collection of co-located squads with less than 100 members and aims to promote collaboration among the squads. Within the same Tribe, there are small groups of people, called Chapters, that work within the same competency area and have similar skill sets. While Chapters are always located within a specific Tribe, there are groups of people, called Guilds, who are wide-reaching with a desire to share knowledge across the whole organisation.

A squad leader is responsible for communicating what problem needs to be solved and why. Squads’ job is to collaborate to find a solution [6]. A Squad has access to a coach, which is responsible for improving squads’ ways of working. Each squad has a Product Owner, who is responsible for prioritising the work, matching product backlog for each squad, and maintaining a high-level roadmap for the organisation.

### Agile Architecture

Iterative and incremental way of architecture evolution is recognised by previous research as an agile way to reduce Big Design Up-Front and to keep a project synchronised with the latest changing conditions [7]. Previous research realised the coexistence of software architectures and agile development in the utilisation of identified architecting activities and approaches, as well as agile architecting practices [13]. Yang et al. [13] identified 41 agile architecting practices. However, only a few of these practices have been widely employed in practice and discussed in the literature – such as Backlog, Sprint, Iterative and Incremental Development, Just Enough Architectural Work, and Continuous Integration.

Neglecting certain architectural considerations even early in the software development process can make architectural refactoring costly [2]. What teams build is influenced and constrained by how they build it. Yet, how teams build something is affected and also constrained by their design and architecture [2]. Hence, agile practitioners need to focus on *“what architectural issues block a team’s agility”* to achieve technical excellence, good design and improve the agility of software development [2]. For instance, modular architecture and microservices are identified as prerequisites for applying agile practices [5].

## Research Design and Methodology

Our case study is carried out in a multinational FinTech organisation that employs around 650 people in 60 markets. This organisation processes around 60 billion  per year. Our case study project is considered as an offshore outsourced FinTech project, which manages hundreds of autonomous financial services. The development programme of our case study project is of large-scale size (<100 people). The developers are distributed over 6 squads. Also, there is 1 Architect, 3 Key Account Managers, 5 Product Owners, 2 Agile Coaches, and 1 Test Lead.

The intervention embedded case-study was conducted in one squad and two Chapters within the case study organisation. This squad consists of 6 developers – 2 of them are Chapter Leaders. The data were collected through direct observation of agile practices for 9 weeks, during which 23 ceremonies were observed. After the intervention trial, 6 semi-structured open-ended interviews were conducted and continued for around 50 min. After the second interview, the questions were revised. After conducting each interview, the recording was transcribed verbatim and analysed in a continuous basis.

The collected data was analysed using Thematic Analysis [1] and informed by some Grounded Theory techniques [3]. Our analysis was carried out by following the six steps proposed by Braun and Clarke [1]: (1) familiarising with the data, (2) generating initial codes, (3) searching for themes, (4) themes review and refinement, (5) defining and naming themes, and (6) writing the final report. During these steps, we utilised some Grounded Theories techniques such as continuous memoing, open coding, constant comparison, and sorting [3]. Furthermore, the observations were analysed and compared to the derived themes from the analysed interviews. In result, minor contradictions were identified, which were explored and accommodated accordingly.

## Findings

This section presents the findings of our study, before and after conducting the intervention embedded case study. Also, this section describes the characteristics of our introduced architecture governance approach, which incorporates an organisational structural change and a change management process. Moreover, this section describes the reported benefits and challenges of the evaluated approach.

### Before Conducting the Intervention – Baseline

Before starting this intervention embedded case-study, our case-study organisation was utilising Spotify’s organisational structure while exercising a centralised based architectural decision-making because of the complexity of this FinTech project. Practitioners say: * “Despite having chapters communities, I was the main reference for all squads when it comes to any architectural based change because of the complexity of the project”*–P2, Enterprise Architect. Also, * “we (developers) were always turning to our architect when it comes to architectural based decisions to figure out the best way to perform an architectural change”*–P4, Senior Developer and Chapter Leader. However, our case study organisation had challenges in aligning and governing architectural decisions across autonomous squads. *“The size of the development programme is now much larger than what it was 3 years ago... I’m overloaded with many responsibilities, which in turn causes a delay in taking architectural decisions and impacts squads autonomy”*–P2, Enterprise Architect.

### After Conducting the Intervention – The Evaluated Approach

**Organisational Structural Change:** This intervention introduced a change to the organisational structure. This change aims to facilitate the alignment architectural decisions across autonomous squads and ultimately to strengthen the autonomy of squads. The structural change is presented in (1) empowering Chapter Leaders and other developers with the role of Architecture Owners, (2) changing the responsibilities of the architect to be of Enterprise Architectural focus, and (3) locating all Architecture Owners in a virtual squad that is led by an Enterprise Architect.

The role of *Architecture Owners* is assigned to Chapter Leaders. Since Chapters are formed based on competency areas, and Squads are aligned on the product-level, the Architecture Owners were aligned accordingly. Practitioners say: * “Giving me the role of Architecture Owner facilitates taking architectural decisions within my Chapter”*–P4, Senior Developer and Chapter Leader. Also, * “breaking down the role of the architect into Architecture Owners roles and distribute it among Chapter Leaders, based on their competency areas transforms decisions into the operational level, which is beneficial in aligning architectural based decisions”*–P1, Agile Coach.

The role of *Enterprise Architect* is assigned to the architect. The architect’s responsibilities are changed to be of enterprise nature. Practitioners say:* “The architect has great knowledge about the technical and the business roadmaps of our organisation... He should continue focusing on the Enterprise architectural tasks”*–P1, Agile Coach. This Enterprise Architect should support and help Chapter Leaders in tackling architectural based decisions. A practitioner says: * “It is vital to have the required commitment and support from our the Enterprise Architect in taking enterprise architectural decisions such as integrating two intercorrelated components or even specifying how to expose some APIs”*–P5, Senior Developer and Chapter Leader.

A virtual architecture squad, which consists of Architecture Owners and Enterprise Architect, was created to facilitate the technical and architectural governance and alignment among autonomous squads. Architecture Owners should have “willing” to collaborate closely with the Enterprise Architect and other Architecture Owners. This is to get the best out of the Architecture Squad and to utilise better alignment across the organisation. A practitioner says: * “The main reason behind creating this virtual architecture squad is to have proper technical and architectural based alignment through the organisation... Meeting whenever needed is important to resolve encountered obstacles”*–P1, Agile Coach.

**Change Management Process:** Our introduced change management process was adapted throughout the intervention trial. This evaluated change management process aimed to guide the involved stakeholders – including the developers, Architecture Owners, Enterprise Architects and Product Owners – in governing and aligning architectural based decisions. This process is comprised of those activities illustrated in the figure shared online in [10].

When a developer encounters a possible architectural change, the developer will determine the impact of the architectural change, create a Kanban card describing the change request and visualise it as a WIP in the analysis phase. Then, the architecture owner and the involved developers should understand the nature of the change and determine its potential architectural impact. The Architecture Owner updates the Kanban card with more accurate technical specifications. If the work requires an enterprise architectural change, the architecture owner should discuss the required change with the enterprise architect and if needed within the architecture squad. An iterative impact analysis process can be conducted based on the encountered challenges. In case of identifying newly impacted components, the architecture owner will create new user story for unpredicted changes. If the change request was approval by the architecture squad and the architecture owner, the Kanban card should be available for the planing and development. Consequently, POs can plan the implementation of this change request and forward the user story and its tasks to the relevant squads for implementation. The squads utilise a hybrid process of Behaviour Driven Development and Test Last Development. Also, the developers utilise the continuous integration to avoid delays caused by integration problems. Also, the scope of testing is extended from test cases to behaviour requirement. Based on the testing results, a new release can be planned for deployment on production.

### Benefits and Challenges of the Evaluated Approach

The practitioners in our case study reported benefits of this introduced approach. Firstly, it has shifted the boundaries and transformed the architectural based decisions into decentralised decision-making. A practitioner says: * “I do not need to wait for the architect anymore... Instead, I can get in touch directly with our Chapter Leader (Architecture Owner)”*–P6, Senior Developer. However, enterprise architectural decisions need to be discussed within the architecture squad. A practitioner says:* “Taking decisions about how to integrate different components, or APIs might require a deep investigation by multiple Architecture Owners and the Enterprise Architect”*–P4, Senior Developer and Chapter Leader. Secondly, our approach has facilitated resolving conflicted architectural decisions and mitigating key technical risks across autonomous squads. Developers might encounter conflicted architectural decisions and not always come to an agreement. A practitioner says:* “Many developers are smart and strong-willed where they do not always come to an agreement... Someone should lead and facilitate the evolution of the architecture”*–P4, Senior Developer and Chapter Leader. Thirdly, our approach has facilitated sharing architectural knowledge among the squads. A practitioner says: * “Our Enterprise Architect started arranging and conducting workshops to train and coach our squads in architectural related aspects”*–P4, Senior Developer and Chapter Leader. Fourthly, our approach has improved software quality and mitigated obstacles to aligning architectural decisions across autonomous squads. Practitioners say:* ‘Conducting proper architectural analysis within our Chapter and then evaluating and discussing the results, if needed, with the Enterprise Architect improves the quality of our produced work”*–P4, Senior Developer. Yet,* “overlooking some aspects that can be considered at the time being might cause a lot of waste because of the need for refactoring”*–P6, Senior Developer.

The practitioners in our case study reported a challenge of this introduced approach. This challenge is presented in prioritising user stories without considering the technical and architectural aspects, which can in turn impact the planning activity negatively. The introduced change management process does not support a process for screening the user stories by the Architecture Squad before conducting the planning. A practitioner says: * “Right now, we do not go through the user stories, in our Architecture Squad, before planning... Yet, sometimes we discuss them informally upon POs request”*–P5, Senior Developer and Chapter Leader. However, the introduced change management process handles such situations when discovering unpredicted architectural changes. This is achieved by moving from Step 6 to Step 1, as illustrated in [10]. The case study organisation considers a spike as an investment to figure out what needs to be built and how. A practitioner says:* “We allocate some resources for complicated work items, ahead of the targeted delivery deadline, to find out what needs to be done... Such investments are considered as necessity to solve architectural issues, which work as enabler for the next Sprint”*–P3, Product Owner.

## Discussion and Conclusion

The topic of autonomous teams is immature within software engineering since there are challenges that need to be addressed [12]. One of these identified challenges that need exploration is how system architecture can best support the coordination of autonomous teams [12]. Our previous research on the Spotify model has revealed a novel approach to agile tailoring, which we called Heterogeneous Tailoring [11]. One of our identified challenges to the Heterogeneous Tailoring approach is the need for aligning and governing architectural decisions across autonomous squads [11].

We conducted an intervention embedded case study to overcome the challenge of aligning architectural decisions across autonomous squads. In this intervention, we developed and evaluated an approach to agile architectural governance, which comprises a structural change and a change management process.

Our findings demonstrate that team-external (i.e., architect) influence over architectural based decisions is negatively related to teamwork quality and team autonomy. The external dependencies to one person decrease team autonomy and lead to a bottleneck since one person bears many responsibilities and has to reply to many different demands simultaneously. In fact, team-external dependencies to individuals should carefully consider any interference with operational project decisions since it is negatively related to important collaborative processes in the teams [4, 11]. Therefore, we have introduced Architecture Owners roles within Chapters to devolve architecture decision making to the operational level. Also, we have changed the responsibilities of the Architect to be of Enterprise Architectural focus to facilitate enterprise architecture decision making, resolve conflicted architectural decisions, and mitigating key technical risks across autonomous squads.

Our evaluated change management process comprises a set of activities, which cover 7 activities out of 11 that have been identified by Yang et al. [13]. These activities are Architectural Analysis and Synthesis (Activity 1 and 2), Architectural Evaluation and Impact Analysis (Activity 3), Architectural Refactoring (Activity 6), and Architectural Maintenance and Evolution (from Activity 6 back to Activity 1). However, Architectural Description and Understanding are used to some extent at the enterprise level. In addition, Architectural Reuse is observed within the squads and encouraged by Architecture Owners.

In this paper, we identified the characteristics and benefits of our evaluated approach to Agile architectural governance using the Spotify model. Also, we identified guidelines and challenges for those wishing to adopt this approach.
